# RNA STRAND: The RNA Secondary Structure and Statistical Analysis Database

**DOI:** 10.1186/1471-2105-9-340

**Published:** 2008-08-13

**Authors:** Mirela Andronescu, Vera Bereg, Holger H Hoos, Anne Condon

**Affiliations:** 1 Department of Computer Science, University of British Columbia, 2366 Main Mall, Vancouver, B.C., Canada

## Abstract

**Background:**

The ability to access, search and analyse secondary structures of a large set of known RNA molecules is very important for deriving improved RNA energy models, for evaluating computational predictions of RNA secondary structures and for a better understanding of RNA folding. Currently there is no database that can easily provide these capabilities for almost all RNA molecules with known secondary structures.

**Results:**

In this paper we describe RNA STRAND – the RNA secondary STRucture and statistical ANalysis Database, a curated database containing known secondary structures of any type and organism. Our new database provides a wide collection of known RNA secondary structures drawn from public databases, searchable and downloadable in a common format. Comprehensive statistical information on the secondary structures in our database is provided using the RNA Secondary Structure Analyser, a new tool we have developed to analyse RNA secondary structures. The information thus obtained is valuable for understanding to which extent and with which probability certain structural motifs can appear. We outline several ways in which the data provided in RNA STRAND can facilitate research on RNA structure, including the improvement of RNA energy models and evaluation of secondary structure prediction programs. In order to keep up-to-date with new RNA secondary structure experiments, we offer the necessary tools to add solved RNA secondary structures to our database and invite researchers to contribute to RNA STRAND.

**Conclusion:**

RNA STRAND is a carefully assembled database of trusted RNA secondary structures, with easy on-line tools for searching, analyzing and downloading user selected entries, and is publicly available at .

## Background

**Figure 1:**
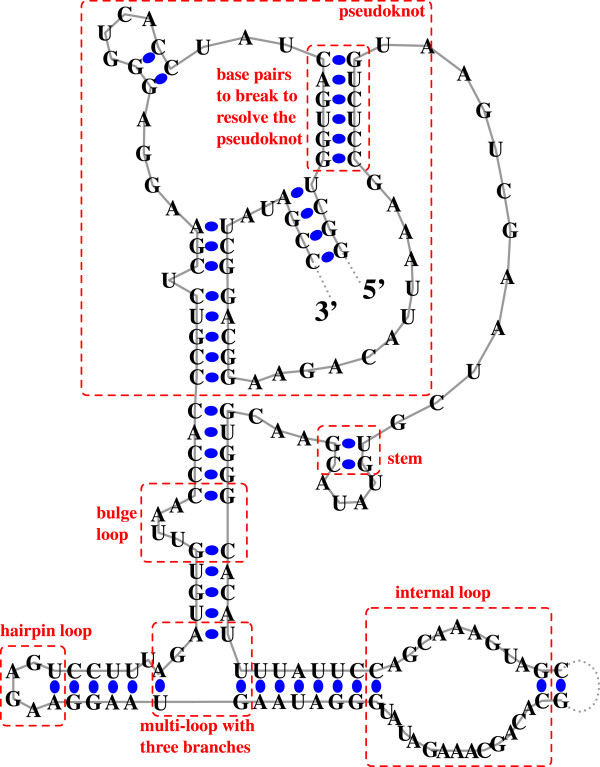
**RNA secondary structure example**. Schematic representation of the secondary structure for the RNase P RNA molecule of *Methanococcus marapaludis *from the RNase P Database; the RNA STRAND ID for this molecule is ASE_00199. Solid grey lines represent the ribose-phosphate backbone. Dotted grey lines represent missing nucleotides. Solid circles mark base pairs. Dashed boxes mark structural features. We define an RNA secondary structure as a set of *base pairs *[22]. In this work, we consider all C-G, A-U and G-U base pairs as *canonical*, and all other base pairs as *non-canonical*. However, we note that from the point of view of the planar edge-to-edge hydrogen bonding interaction [42], there are C-G, A-U and G-U base pairs that do not interact via Watson-Crick edges, and vice-versa [14,42]. Comparative sequence analysis tools do not currently describe bond types. A number of structural motifs can be identified in a secondary structure: A *stem *is composed of one or more consecutive base pairs. A *hairpin loop *contains one closing base pair, and all the bases between the paired bases are unpaired. An *internal loop *is a loop with two closing base pairs, and all bases between them are unpaired. A *bulge loop *can be seen as a variant of an internal loop in which there are no unpaired bases on one side. A *multi-loop *is a loop which has at least three closing base pairs; stems emanating from these base pairs are called *multi-loop branches*. A *pseudoknot *is a structural motif that involves non-nested, crossing base pairs.

The number of solved RNA secondary structures has increased dramatically in the past decade, and several databases are available to search and download specific classes of RNA secondary structures [1-5]. However, for purposes such as improving RNA energy models [6,7], evaluating RNA secondary structure prediction software, obtaining distributions of naturally occuring structural features, or searching RNA molecules with specific motifs, researchers need to easily access a much larger set of known RNA secondary structures, ideally all known RNA secondary structures. RNA STRAND aims to provide this capability, in addition to easy search, analysis and download features. Figure 1 shows an example of an RNA secondary structure and highlights some of its structural features.

Previous RNA databases provide secondary structure information, but are specialised in a different direction or follow different goals. The Rfam Database [5] contains a large collection of non-coding RNA families; however, many of the corresponding secondary structures are computationally predicted. The Comparative RNA Web Site [1] specialises in ribosomal RNA and intron RNA molecules. The Sprinzl tRNA database [2] specialises in tRNA molecules, the RNase P database [3] specialises in RNase P RNA molecules, and the SRP and tmRNA databases [4] specialise in SRP RNA and tmRNA molecules, respectively. Pseudobase [8] contains short RNA fragments that have pseudoknots. The RAG (RNA-As-Graphs) Database [9] classifies and analyses RNA secondary structures according to their topological characteristics based on the description of RNAs as graphs, but its collection of structures is very limited.

A number of previous databases contain three-dimensional (3D) RNA structures; however, as opposed to proteins, the number of solved RNA 3D structures is much smaller than the number of solved RNA secondary structures. (Only 18% of all RNA molecules we collected have known 3D structures.) As such, all these databases do not include molecules whose secondary structures are known but 3D structures are unknown; examples include: the RCSB Protein Data Bank [10], the Nucleic Acids Database [11], the RNA Structure Database [12] and the Structural Classification of RNA (SCOR) database [13]. NCIR [14] contains non-canonical base pairs in 3D RNA molecules. FR3D [15] provides a collection of 3D RNA structural motifs found in the RCSB Protein Data Bank. Finally, there are other RNA databases that provide RNA sequences, but no experimental structural information, such as the SubViral RNA Database [16], which contains a collection of over 2600 sequences of viroids, the hepatitis delta virus and satellite RNAs, but only mfold-predicted secondary structures.

RNA STRAND spans a more comprehensive range of RNA secondary structures than do previous databases. It currently provides highly accurate secondary structures for 4666 RNA molecules. Since some users of RNA STRAND will likely develop new thermodynamic models, prediction tools or statistical analyses, our data is exclusively determined by carefully conducted comparative sequence analysis [1], or by experimental methods such as NMR or X-ray crystallography [10]. All information has been obtained from publicly available RNA databases. Our goal in creating this database is to provide comprehensive information on structural features – such as types and sizes for stems and loops, pseudoknot complexity and base pair types – that can be interactively analysed or downloaded within and across functional classes of molecules. Such information could be used, for example, to understand what type of structural motifs are common in a specific set of RNA molecules; to estimate the accuracy of RNA secondary structure computational prediction methods; or to improve current thermodynamic models for RNA secondary structure prediction.

## Construction and Content

**Figure 2:**
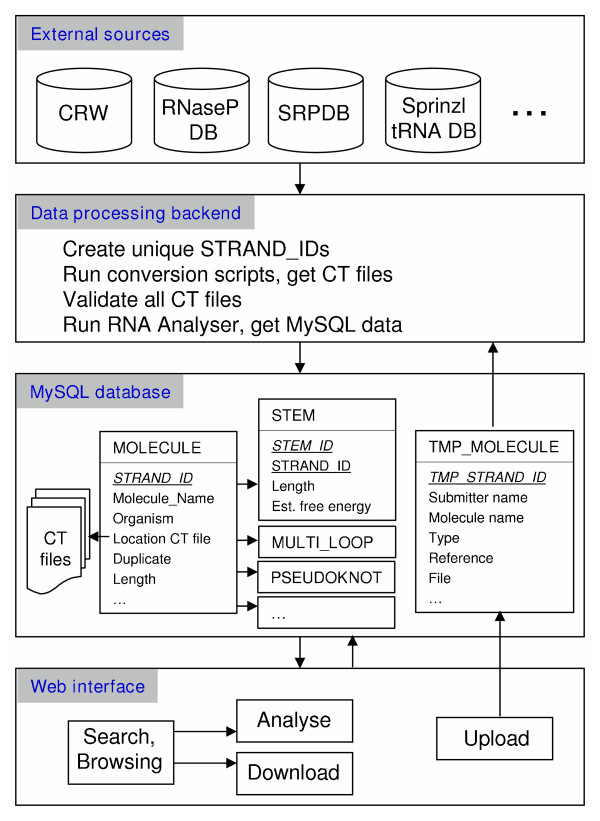
**Database schema**. Construction of RNA STRAND, from data collection to data presentation via dynamic web pages.

Figure 2 describes the four main modules that comprise RNA STRAND. To create the database, we first collected the data from various external sources, then we processed the data and prepared it for a MySQL relational database. Next, we installed and populated the database, and finally we prepared dynamic web pages that interact with the database. In what follows we describe in detail the construction and content of each module.

### External sources

The current release v2.0 of RNA STRAND contains a total of 4666 entries (RNA sequences and secondary structures) of the following provenance:

• RCSB Protein Data Bank (PDB) [10]: 1059 entries, obtained from three dimensional NMR and X-ray atomic structures containing RNA molecules only, or RNA molecules and proteins (only the RNAs were included in RNA STRAND), in PDB format. These include ribozymes, ribosomal RNAs, transfer RNAs, synthetic structures, and complexes containing more than one RNA molecule. Out of the 1059 entries, 575 contain at least two RNA molecules; these are easily searchable from the RNA STRAND web site. The RNA secondary structures were generated from the tertiary structures using RNAView [17], which is also used for secondary structure visualisation in the Nucleic Acid Database [11].

• Comparative RNA Web Site, version 2 [1]: 1056 entries of ribosomal and intronic RNA molecules obtained by covariance-based comparative sequence analysis.

• tmRNA database [4]: 726 entries of transfer messenger RNA sequences and secondary structures determined by comparative sequence analysis.

• Sprinzl tRNA Database (September 2007 edition) [2]: 622 transfer RNA sequences and secondary structures obtained by comparative sequence analysis from the tRNA sequences data set. The genomic tRNA and tRNA gene sets from the Sprinzl tRNA database contain genomic sequences, and thus we think they are not as relevant for understanding function and folding of functional RNA molecules.

• RNase P Database [3]: 454 Ribonuclease P RNA sequences and secondary structures obtained by comparative sequence analysis.

• SRP Database [4]: 383 entries of Signal Recognition Particle RNA sequences and secondary structures determined by comparative sequence analysis.

• Rfam Database, version 8.1 [5]: 313 entries from 19 Rfam families, including hammerhead ribozymes, telomerase RNAs, RNase MRP RNAs and RNase E 5' UTR elements (only the seeds have been used). Of the 607 Rfam families in version 8.1, 172 have the secondary structure flag "published", while the remaining 435 families have been predicted using Pfold [5]. For several reasons, we decided to include only 19 of the 172 "published" families: (1) some of these families come from other databases that we have included directly, such as structures from the RNase P Database or SRP Database; (2) most of the secondary structures are actually predicted computationally and then published in the papers cited by Rfam, such as families RF00013, RF00035, RF00161 or RF00625. Since the Rfam database provides only very limited information about the reliability of the structures it contains, we have studied all 172 families and decided which families to include based on the cited papers. The details regarding the decision for each family are described in Supplementary Material 1, accessible from the main page of the RNA STRAND web site.

• Nucleic Acid Database (NDB) [11]: 53 entries which occur in NDB and not in PDB (note that NDB and PDB have a large overlap of RNA structures); these include transfer RNAs and synthetic RNAs obtained by X-ray crystallography.

Table 1 provides some additional information on these RNAs; information and statistics on the current database contents are also available from the main page of the RNA STRAND site.

**Table 1:** The main RNA types included in RNA STRAND v2.0.

RNA type	Main source(s)	#	Length		% PKBP	
		entries	mean	std	mean	std
Transfer messenger RNA	tmRDB [4]	726	368	86	21.0	6.1
16S ribosomal RNA	CRW [1], PDB [10]	723	1529	286	1.8	0.5
Transfer RNA	Sprinzl DB [2], PDB [10]	707	76	21	0.1	2.3
Ribonuclease P RNA	RNase P DB [3]	470	323	71	5.7	3.2
Signal rec. particle RNA	SRPDB [4], PDB [10]	394	220	111	0.0	0.0
23S ribosomal RNA	CRW [1], PDB [10]	205	2699	716	2.4	1.1
5S ribosomal RNA	CRW [1], PDB [10]	161	115	21	0.0	0.0
Group I intron	CRW [1], PDB [10]	152	563	412	5.8	2.2
Hammerhead ribozyme	Rfam [5], PDB [10]	146	61	24	0.0	0.0
Group II intron	CRW [1], PDB [10]	42	1298	829	1.4	3.5

All molecules	All of the above	4666	527	722	5.3	9.1

Overview of the main RNA types in version 2.0 of the RNA STRAND database, their provenance, the number of RNAs, the mean length and standard deviation for each type. % PKBP denotes the percentage of the base pairs that need to be removed in order to render the structure pseudoknot-free. Most of the major RNA types are represented by a large number of molecules.

In the future, we intend to regularly check the aforementioned databases for new entries. With our current tools, keeping the database up-to-date will be relatively easy.

### Data processing

#### Unique IDs

We created a unique and stable identifier for each entry in the RNA STRAND database. Future releases will keep all previous IDs unchanged.

#### Conversion scripts

One of the challenging tasks in collecting the RNA STRAND data arose from the fact that the external sources offer data in various formats. We have built tools to convert from all these formats to the CT format, which we use to store all structures internally, and to RNAML, BPSEQ, dot-parentheses and FASTA formats when requested by a user. The format descriptions are accessible on the "Help" page online.

#### Validation

All external databases we have used in the current version of RNA STRAND, except Rfam, contain highly curated RNA secondary or tertiary structures, therefore we trust the curation methods of these sources. For Rfam we selected a set of reliable structures based on the cited papers, as described in the previous section. Once we converted all the secondary structure external files into the CT format, we checked all files in order to make sure the secondary structures are valid (i.e., one base is paired with at most one other base, and if base at position *i *is paired with base at position *j*, then base at position *j *is also paired with base at position *i*.). When performed on our present data, this validation step revealed several inconsistencies in some of the external files, which we brought to the attention of the respective database owners.

#### RNA Secondary Structure Analyser

The structural statistics that form the core part of RNA STRAND were generated using the RNA Secondary Structure Analyser, which takes as input an RNA secondary structure description, for example in CT format, and outputs a wide range of secondary structure information. While many of these features, such as the number and composition of stems, are rather straightforward to determine, in some cases, more advanced algorithmic techniques have to be applied – as is the case, for example, for the minimal number of base pairs that need to be removed to render a structure pseudoknot free. For this specific task, we implemented a dynamic programming algorithm that removes the minimum number of base pairs [18]; however, more sophisticated approaches could be used, such as those recently described by Smit et al. [19]. The complete output of the analyser run for each individual database entry can be accessed easily from the RNA STRAND web interface, and a description of the output can be found in the online Supplementary Material 2.

### MySQL database

All the data obtained from the RNA Secondary Structure Analyser were inserted into a relational database implemented in MySQL (version 5.0.26). The main table is MOLECULE, with one row per RNA entry in the database. This table contains as primary key the unique RNA STRAND ID of the entry and further comprises various descriptive fields, including: organism, reference, length, RNA type, external source, external ID, sequence, three levels of abstract shapes using the RNAshapes representation [20], the method of secondary structure determination, and a link to the respective CT file. (Since RNAshapes version 2.1.5 cannot obtain the abstract shape of pseudoknotted secondary structures, we first removed a minimum number of base pairs to render the structure pseudoknot-free.) Furthermore, there is one table per secondary structure feature, where the table MOLECULE is connected to each of these tables in a one-to-many relationship. For example, the table STEM contains information such as the number of base pairs and the estimated free energy change for that stem, using parameters by Xia et al. [21]. Accurately estimating the free energy change of entire structures is currently challenging, due to structural motifs for which current energy models are incomplete, such as pseudoknots, non-canonical base pairs, and modified nucleotides. Other similar tables include HAIRPIN_LOOP, MULTI_LOOP and PSEUDOKNOT.

An additional table TMP_MOLECULE is used to temporarily hold new submissions received via the web interface; for these, we manually check the submission information by checking the cited paper, after which, if the submission is accepted, all further steps required to permanently add the respective RNA(s) to the database are performed automatically.

### Web interface

The web interface to RNA STRAND has been created using a set of PHP scripts (version 5.1.2). The main functions of the web interface are searching, browsing, analysis, downloading and uploading.

#### Searching and browsing

The user specifies one or more search criteria in a web-based form. The general criteria include RNA type (e.g., 16S Ribosomal RNA), organism of origin (e.g., *E. coli*), external source (e.g., RCSB Protein Data Bank), length (in bases), the number of molecules in the complex, whether it is a fragment, a sequence pattern using the standard IUPAC nucleic acid codes, an abstract structure or fragment using the RNAshapes representation [20] and whether or not to include non-redundant sequences.

We define a set of entries to be non-redundant if their sequences are pairwise distinct. On a search page the user can request a non-redundant set that satisfies some search criteria. In this case, if two entries have identical RNA sequences, one of them will be selected arbitrarily. In the remainder of this paper, when we refer to a number of non-redundant entries matching some criteria, we mean a largest non-redundant set of entries satisfying the specified criteria. Currently there are 4104 non-redundant entries out of the 4666 entries in RNA STRAND v2.0.

Advanced searches are supported based on 21 additional search criteria on secondary structure elements, such as selection of RNA molecules having at least one pseudoknot, or hairpin loops with a specific sequence – for example GNRA hairpin loops. The set of database entries that match all of the specified criteria simultaneously is returned in the form of a table.

Using advanced search criteria, users can search for entries with various structural motifs. For example, when looking for a Y shape with an additional hairpin, one would search for entries that have exactly one multi-loop, three multi-loop branches, three hairpins, one molecule in the complex, and no pseudoknots. This search returns 31 entries, most of which are *ciliate telomerase RNAs *from Rfam. If pseudoknots are allowed, then *vertebrate telomerase RNAs *from Rfam are also included, yielding 36 search results. An equivalent pseudoknot-free search can be obtained by typing in the abstract shape [ [] [] ] [] (where matching brackets represent one interrupted or uninterrupted stem). Pseudoknots are currently not permitted in the abstract shape representation [20].

Support for inspecting large fractions of the database contents is provided via searches with no or very general criteria. For example, it is easy to obtain a list of all RNase P RNA structures contained in the database.

Details on individual entries from the result list of any search can be displayed by clicking on an RNA STRAND ID link of the results table. This single entry display comprises general information about the entry, links to the original database entry for this molecule, a secondary structure diagram, details of its secondary structure elements and features, links to other RNA STRAND entries with the same sequence (i.e., redundant entries), links to the sequence and secondary structure specification in five formats (CT, RNAML, BPSEQ, dot-parentheses and FASTA), and a link to the complete output of the RNA Secondary Structure Analyser.

#### Analysis

In addition to the aforementioned analysis information for individual entries, RNA STRAND also provides histograms or cumulative distribution functions of various molecule characteristics (such as number of pseudoknots per molecule) or structural features (such as number of branches per multi-loop) for all structures in the database or for user-selected subsets, as obtained from the search page. In addition, correlations between various molecule characteristics and molecule length can be obtained. For an unbiased analysis, the user has the option of normalising the data by RNA type (such as tRNA), in which case for each particular RNA type, one data point is obtained by averaging over all the data for molecules of that type. Finally, the user can choose to remove the outliers of the distributions. We use a common definition, according to which a data point is an outlier if, and only if, it is smaller than *Q*_1 _- 1.3·(*Q*_3 _- *Q*_1_) or greater than *Q*_1 _+ 1.3·(*Q*_3 _- *Q*_1_), where *Q*_1 _and *Q*_3 _are the first and third quartiles, respectively. Such analyses may guide research pertaining to understanding structural features in naturally occuring RNA molecules, as we outline in the "Utility and discussion" section.

#### Downloading

The set of molecules selected via the search page can be downloaded in one of five supported formats: CT, RNAML, BPSEQ, dot-parentheses and FASTA. Thus, researchers can use specifically selected structures locally.

#### Uploading

RNA STRAND supports public submission of RNA secondary structures to the database via its web interface. The structure file can be in any of the four supported secondary structure formats (CT, RNAML, BPSEQ and dot-parentheses) or in the PDB tertiary structure format. Since RNA STRAND is a curated database, newly submitted structures are checked for accuracy and completeness by one of the database administrators before they are added to the database. New additions to the public databases that constitute our external sources will be added to RNA STRAND regularly. This is complemented by the public submission option, which is intended for submission of structures that do not yet belong to any of these databases.

### Utility and discussion

RNA STRAND v2.0 contains 4666 RNA molecules or interacting complexes of various types, and an abundance of RNA structural motifs (see also Table 1). This represents a considerable amount of data from which to draw significant statistics and trends about RNA secondary structures. from which to draw significant statistics and trends about RNA secondary structures. In what follows we illustrate how the information in RNA STRAND can be used for various purposes.

### Obtaining statistics of naturally occuring RNA structural features

We performed statistical analyses using the RNA STRAND web interface. Our first observation concerns the number and complexity of pseudoknots. According to the current data from RNA STRAND v2.0, pseudoknots occur rather commonly, especially in longer molecules: 74% of all (non-redundant) entries with 100 or more nucleotides contain pseudoknots. We compared the stem length (i.e., the number of base pairs in uninterrupted stems) with the minimal number of base pairs that need to be removed per pseudoknot to render the structure pseudoknot free (we denote this number by # PKBP; note that for over 95% of the pseudoknots, the bases counted in determining # PKBP form one uninterrupted stem; also, there is no overlap between the base pairs counted in determining the stem length and the base pairs counted in determining # PKBP). Table 2 shows that when considering all RNA types in the database, the median, mean and standard deviation of the two measures, stem length and # PKBP, are very similar, even when we normalise by RNA type. (For normalised analysis, instead of using one data point per molecule or per structural feature, we use one data point for each RNA type, where this point is determined by averaging all data points for the respective class of RNAs. This way, the user can avoid biasing the analysis when there are substantially more structures for some RNA types than for others.) However, for 16S and 23S ribosomal RNA molecules the stem length tends to be significantly larger than # PKBP, whereas for transfer messenger RNA molecules in particular and ribonuclease P RNA molecules to some extent, # PKBP is larger than the stem length. This observation is interesting in the context of computational approaches for RNA secondary structure prediction which ignore pseudoknots [22], add pseudoknots hierarchically in a second stage [23], or simultaneously add stems in pseudoknotted and non-pseudoknotted regions [24,25].

**Table 2:** Statistics on the complexity of pseudoknots in RNA STRAND v2.0.

RNA type	#	Stem length			# PKBP		
		
	entries	median	mean	std	median	mean	std
16S ribosomal RNA	644	4.00	4.30	2.50	3.00	2.50	0.68
23S ribosomal RNA	93	4.00	4.14	2.39	2.00	3.75	3.12
Transfer messenger RNA	657	4.00	4.11	2.24	5.00	5.51	1.00
Ribonuclease P RNA	433	4.00	4.45	2.51	4.00	5.18	1.36

All, non-redundant	4104	4.00	4.35	2.44	4.00	4.14	1.86
All, non-redundant & normalised	4104	4.96	5.05	0.58	4.65	4.95	1.78

The columns represent the RNA type, the number of entries for each type, the median, mean and standard deviation of the stem length (i.e., number of adjacent base pairs) and the minimum number of base pairs to break in order to open pseudoknots (# PKBP). For each row, a non-redundant set was selected, and outliers were removed.

**Figure 3:**
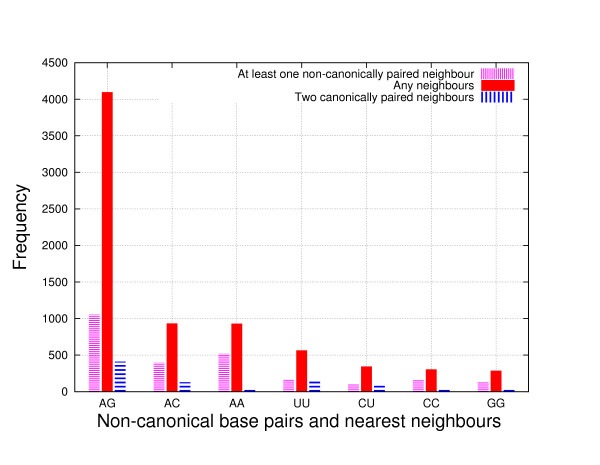
**Histogram of the occurence of non-canonical base pairs**. Histogram of non-canonical base pairs in the 729 non-redundant entries whose structures were determined by NMR or X-ray crystallography.

Our second observation concerns the abundance of non-canonical base pairs and the pairing type of their immediate neighbours. (We consider all C-G, A-U and G-U pairs to be *canonical base pairs*, and all other base pairs to be *non-canonical*.) Figure 3 shows a histogram for the 729 non-redundant entries whose structures were determined by all-atom methods (these include structures from the Protein Data Bank and the Nucleic Acid Database). For this data set, non-canonical A-G base pairs are the most abundant, representing 55% of all non-canonical base pairs, and G-G pairs are the least abundant, representing only 4% of all non-canonical base pairs. The plot also shows that a relatively small fraction of non-canonical base pairs have as immediate neighbours canonical base pairs. Interestingly, for all seven types of non-canonical base pairs, more pairs are adjacent to at least one other non-canonical base pair than surrounded by two canonical base pairs. For example, 55% of all A-A pairs are adjacent to at least one other non-canonical base pair. This may suggest that non-canonical base pairs are sufficiently stable energetically to form several consecutive base pairs.

Finally, we found rather strong linear correlations between the number of nucleotides of the RNAs in our database and the number of stems, hairpin loops, bulges, internal loops and multi-loops; the Pearson correlation coefficients are *r *= 0.95, 0.95, 0.92, 0.91 and 0.92, respectively. This is consistent with the idea that the local formation of these secondary structure elements is relatively independent of the overall size of the molecule and in agreement with the current thermodynamic energy models of RNA secondary structure, which assume additive and independent energy contributions for these structural elements. Interestingly, the correlation between the RNA length and the number of pseudoknots is significantly weaker (*r *= 0.64), suggesting that pseudoknots may not follow the same linearity principle.

### Evaluating energy-based secondary structure prediction programs

The RNA STRAND database can be used to evaluate the prediction accuracy of energy-based RNA secondary structure prediction software. RNA STRAND v2.0 contains 3704 non-redundant entries containing one molecule that can be used to evaluate software such as CONTRAfold [7] or mfold [26], 403 non-redundant entries containing complexes of two or more molecules that can be used to evaluate sofware for interacting molecules [27,28], and 1957 non-redundant single-molecules with pseudoknots that can be used to evaluate secondary structure prediction programs with pseudoknots [23-25,29].

**Figure 4:**
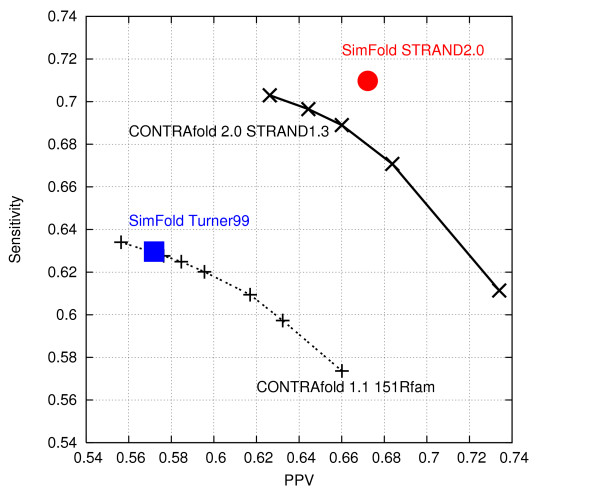
**Prediction accuracy achieved by various energy models**. Sensitivity vs. positive predictive value (PPV) of various secondary structure prediction methods. Sensitivity is the number of correctly predicted base pairs divided by the number of base pairs in the reference structure, PPV is the number of correctly predicted base pairs, divided by the number of predicted base pairs. Higher prediction accuracy is achieved when the free energy parameters are obtained by training on a larger set of structures. The CONTRAfold prediction program uses a trade-off parameter *γ *between sensitivity and PPV, and thus we report predictions for *γ *ranging from 2 to 20.

We have selected 2518 structures out of the 3704 non-redundant entries containing one molecule, after we eliminated the entries with unknown nucleotides and overly large loops. (Specifically, entries having hairpin loops, bulges, internal loops or multi-loops with more than 50, 50, 50 and 100 unpaired bases, respectively, were removed.) In addition, we have removed all non-canonical base pairs and the minimum number of base pairs needed to render the structures pseudoknot-free. The resulting structures are used as ground-truth reference structures. We evaluated the sensitivity and positive predictive value (PPV) of CONTRAfold [7] and SimFold [30] with various free energy parameter sets, see Figure 4. Sensitivity is the number of correctly predicted base pairs divided by the number of base pairs in the reference structure, and PPV is the number of correctly predicted base pairs divided by the number of predicted base pairs. "SimFold Turner99" in Figure 4 refers to SimFold using the free energy parameters described by Mathews et al. [22], and is essentially equivalent to mfold 3.1 [26]. On this large set, the average sensitivity of prediction is 0.63, while the average PPV is 0.57.

"CONTRAfold 1.1 151Rfam" is the CONTRAfold software version 1.1, as reported by Do et al. [7]. The CONTRAfold prediction program uses a trade-off parameter *γ *between sensitivity and PPV, and thus we report predictions for *γ *ranging from 2 to 20. When the target of one measure is fixed to the value obtained with "SimFold Turner99", the other is similar as well, showing that on this data set, CONTRAfold 1.1 gives similar average prediction accuracy as "SimFold Turner99". The remaining points of Figure 4 are described in the following section.

### Improving RNA energy models

More importantly, RNA STRAND can facilitate approaches for improving the free energy models underlying energy-based RNA secondary structure prediction software [6,7]. In this context, it can be very useful to exploit training data consisting of RNA sequences with known secondary structures, and the size and variety of such data are key for obtaining good results.

Figure 4 shows the average sensitivity and PPV of various programs measured on the 2518 structures mentioned in the previous section, and trained on various training sets.

"CONTRAfold 1.1 151Rfam" was trained on a small set of 151 structures from various Rfam families [7], while "CONTRAfold 2.0 STRAND1.3" was trained on 3427 pre-processed structures (i.e., split and restricted) of average length 178 nucleotides from version 1.3 of the RNA STRAND database, as used by Andronescu et al. [6]. The figure shows that using the much larger set in the latter case gives an improvement of roughly 7% in prediction accuracy.

To demonstrate even further the importance of using a large and comprehensive set of known RNA secondary structures for obtaining high-quality free energy parameters, we have used the current version of RNA STRAND v2.0 to obtain a new training set of 2246 structures of average length 246 nucleotides. Using the Maximum Likelihood parameter estimation method described by Andronescu et al. [6], which is similar to CONTRAfold [7], we have improved the average accuracy of prediction even further, as shown by the data point labelled "SimFold STRAND2.0" in Figure 4. This gives an improvement of 8% in average sensitivity and 10% in average PPV compared to the Turner99 parameters, when measured on our test set of 2518 structures. (Note that, since CONTRAfold and SimFold use different energy models and prediction algorithms, it is more appropriate to make comparisons between different versions of each, than it is to compare CONTRAfold versus SimFold).

These results provide clear evidence for the key role of large and carefully assembled sets of RNA secondary structures, such as provided by RNA STRAND, in the context of determining RNA free energy models. In the future, we are planning to use the RNA STRAND data to train free energy parameters for pseudoknotted structures. Existing energy models for RNA secondary structure prediction methods with pseudoknots are often ad-hoc [25,29], and we believe that by using data-driven methods in conjunction with the 1957 non-redundant RNA STRAND entries representing RNAs with pseudoknots, it will be possible to obtain improved energy models for pseudoknotted structure prediction.

### Other uses of RNA STRAND

The numerous search criteria supported by the RNA STRAND web interface allow users to select and study molecules with specific structural features. For example, Tyagi and Mathews [31] studied the computational prediction accuracy of helical coaxial stacking in multi-loops. RNA STRAND v2.0 conveniently allows the selection and download of 189 non-redundant entries with all-atom structures that have at least one multi-loop. Other examples include the use of naturally occuring pseudoknotted structures that can be used to evaluate computational methods to render a pseudoknotted RNA secondary structure pseudoknot free [19], or to evaluate RNA secondary structure visualisation tools [32].

In recent work on the role of RNA structure in splicing, Rogic et al. [33] needed to identify thermodynamically stable stems that maximally shorten the distance between mRNA donor sites and branchpoint sequences. Since the optimal free energy of such stems is unknown, Rogic et al. wished to determine the most probable ranges of possible free energies for uninterrupted stems. By selecting all molecules on the RNA STRAND web site, they obtained distributions of estimated stem free energies (obtained with parameters by Xia et al. [21]), which were used to support a new model for the role of RNA secondary stucture in mRNA splicing.

In addition, RNA STRAND can facilitate the design of optical melting experiments [21], whose goal is to better understand the thermodynamics of RNA structure formation, and to improve RNA secondary structure prediction accuracy. When designing optical melting experiments, usually a set of known RNA secondary structures is first assembled to determine what types of structural motifs that have not been previously studied appear frequently in naturally occuring RNAs [34,35]. The RNA STRAND web interface, as well as the abundance of reliable RNA structures in the RNA STRAND database, can be very useful in this context. For example, a significant number of multi-loops (16% in all non-redundant RNA STRAND entries) have five or more branches, but, to the best of our knowledge, optical melting experiments only exist for multi-loops with up to four branches [36,37]. Moreover, 30% of the internal loops in all non-redundant RNA STRAND entries have seven or more unpaired bases, and 13% have an absolute asymmetry (i.e., absolute difference between the number of unpaired bases on each side) of at least three, while only limited optical melting experiments exist to cover these cases [38,39].

## Conclusion

In this paper, we presented RNA STRAND, a new database for RNA secondary structure data that provides access to detailed information on known secondary structures as well as statistical analyses of structural aspects of various types of RNAs. We believe that such information will be useful in the context of understanding RNA structure and function; in particular, we expect it to further facilitate the development and evaluation of energy models for secondary structure prediction. Our database is flexible and extensible; it provides a convenient web interface to its major functions and supports searches according to many criteria, including properties of secondary structure elements. The database is publicly accessible and supports the submission of new RNA structures by the research community. We are committed to keeping RNA STRAND up-to-date with new structures that are added to the eight databases of provenance, and we invite submissions of all types of RNA secondary structures, which will help to further expand the database and increase its usefulness.

In the future, we intend to add RNA secondary structures obtained from the SHAPE technique [40,41], and also to provide further search options such as searches by specific structural motifs.

## Availability and requirements

RNA STRAND is publicly available at . The RNA Secondary Structure Analyser, as well as the database tables, are available upon request from the authors.

## Authors' contributions

MA collected the data, implemented conversion and validation scripts, implemented the MySQL database and part of the PHP scripts, performed the statistical analyses and helped to draft the manuscript. VB implemented the vast majority of the PHP scripts and most of the RNA Secondary Structure Analyser. HHH and AC conceived the project, specified the design of the RNA Secondary Structure Analyser, supervised MA's and VB's work, and helped to write the manuscript. All authors checked the accuracy of the database and web interface, read and approved the final manuscript.
